# The association of plasma sortilin with essential hypertension and subclinical carotid atherosclerosis: A cross-sectional study

**DOI:** 10.3389/fcvm.2022.966890

**Published:** 2022-10-12

**Authors:** Xinglin Chu, Rui Liu, Chunli Li, Tao Gao, Yongqi Dong, Yi Jiang, Dazhi Ke

**Affiliations:** ^1^Department of General Practice, The Second Affiliated Hospital of Chongqing Medical University, Chongqing Medical University, Chongqing, China; ^2^Department of Oncology, The Second Affiliated Hospital of Chongqing Medical University, Chongqing Medical University, Chongqing, China; ^3^Institute of Life Sciences, Chongqing Medical University, Chongqing, China; ^4^Department of Gastroenterology, The Second Affiliated Hospital of Chongqing Medical University, Chongqing Medical University, Chongqing, China; ^5^Department of General Practice, Chongqing Emergency Medical Center, Chongqing University Central Hospital, Chongqing, China

**Keywords:** sortilin (SORT1), hypertension, subclinical carotid atherosclerosis, endothelial (dys) function, lipid metabolism

## Abstract

**Background:**

Sortilin, a protein that regulates glucose and lipid metabolism, has recently been linked to cardiovascular diseases (CVDs) such as coronary heart disease and carotid artery stenosis. In this study, we measured circulating sortilin concentrations in essential hypertensive (EH) patients, and evaluated the association between sortilin, hypertension, and subclinical carotid atherosclerosis in hypertensive individuals.

**Methods:**

This cross-sectional study included 336 individuals, including 186 newly diagnosed EH patients and 150 age-and-sex-matched normotensive healthy subjects (NT). Plasma sortilin and adiponectin (ADI) levels were measured using ELISA kits. In the EH group, high-resolution B-mode ultrasound was used to detect the existence of subclinical carotid atherosclerosis (subAS), which was defined as having a carotid intima–media thickness (cIMT) ≥ 1.0 mm and/or plaque on the carotid artery without any clinical manifestations.

**Results:**

Our findings showed that plasma sortilin concentrations ranged from 3.34–11.34 ng/ml for all subjects. Sortilin levels were significantly higher in the EH group than in the NT group (8.10 ± 1.82 ng/ml vs. 6.37 ± 1.52 ng/ml, *P* < 0.001) and were further upregulated in the EH with subclinical carotid atherosclerosis (EH + subAS) group compared to the EH without subclinical carotid atherosclerosis (EH-subAS) group (8.42 ± 1.75 ng/ml vs. 7.79 ± 1.84 ng/ml, *P* < 0.05). In correlation analysis, sortilin was positively correlated with systolic blood pressure (SBP), diastolic blood pressure (DBP), triglyceride (TG), total cholesterol (TC), low-density lipoprotein cholesterol (LDL-C), white blood cell (WBC), endothelin-1 (ET-1), high-sensitivity C-reactive protein (hsCRP), interleukin-6 (IL-6), and cIMT (all *P* < 0.05) and negatively associated with NO and ADI (*P* < 0.001). Multiple linear regression analysis revealed that SBP, LDL-C, and ET-1 were independently associated with plasma sortilin levels. Increased sortilin levels were independently associated with the risk of EH (OR: 1.86, 95%CI: 1.56–2.20, *P* < 0.001) and EH + subAS (OR: 1.33, 95%CI: 1.07–1.66, *P* = 0.011), after adjustment for multiple risk factors. Restricted spline curve showed that elevated sortilin levels increase the odds of having EH.

**Conclusion:**

Elevated sortilin levels are associated with an increased risk of essential hypertension and subclinical carotid atherosclerosis in hypertensive patients.

## Introduction

Hypertension is an epidemic affecting one-third of the world’s adult population, and is the main cause of stroke and cardiovascular diseases (CVDs). It is a complicated condition caused by the interplay of biological and environmental factors, with the main pathophysiologic feature being dysfunction of vascular constriction and relaxation (1). Blood vessels are the main target organ of the pathophysiology of hypertension, and there may be no obvious clinical manifestations in the early stage. Long term hypertension can lead to alterations in vascular structure and function, including endothelial dysfunction and early atherosclerosis. However, the mechanism of essential hypertension and early atherosclerosis caused by hypertension remains unclear.

Sortilin or neurotensin receptor 3, belongs to the vacuolar protein sorting 10 protein (Vps10p) domain family, which acts as a receptor responsible for cell survival, signal transduction, and sorting or trafficking proteins within the cell (2). Recently, multiple studies have found that sortilin is a crucial protein involved in the pathogenesis of cardiovascular and metabolic diseases by regulating insulin resistance, atherosclerosis, lipoprotein metabolism, and vascular calcification (3–5). Elevated serum sortilin levels have been linked to coronary artery disease (CAD), peripheral artery disease (PAD), and carotid artery stenosis (CAS) (6–8). Another study found that sortilin affects vascular endothelial function through deregulation of sphingolipid metabolism and oxidative stress, leading to increased blood pressure (9). However, a recent cohort study showed that sortilin was not associated with coronary artery calcium score, disease severity, and CAD prognosis (10). These inconsistent findings on the relationship between sortilin and CVDs need further investigation. Therefore, we conducted a cross-sectional study to explore the associations of plasma sortilin with hypertension, and subclinical carotid atherosclerosis in newly diagnosed hypertensive patients.

## Materials and methods

### Participants enrolled in the study

We enrolled 336 individuals, including 186 newly diagnosed essential hypertensive (EH) patients and 150 age-and-sex-matched normotensive healthy subjects (NT) recruited from our hospital between 2020 and 2021. None of the subjects received antihypertensive or statin therapy. Patients with an initial diagnosis of hypertension with an SBP higher than 140 mmHg or a DBP higher than 90 mmHg were considered newly diagnosed EH patients. EH patients were stratified into two groups according to their carotid artery conditions: the group with subclinical carotid atherosclerosis (EH + subAS, *n* = 93) and the group without subclinical carotid atherosclerosis (EH-subAS, *n* = 93). Subclinical carotid atherosclerosis, which was defined as having a carotid intima–media thickness (cIMT) ≥ 1.0 mm and/or plaque on the carotid artery without any clinical manifestations (11). Patients who satisfied any of the following criteria were excluded: the current use of antihypertensive and statin medications; malignant hypertension or secondary hypertension; fasting blood glucose (FBG) at least 7.0 mmol/l; diabetes, heart disease, chronic or acute infectious disease, tumor, hepatorenal insufficiency, or respiratory disease.

The study was approved by the Human Research Ethics Committee at The Second Affiliated Hospital of Chongqing Medical University, and informed consent was obtained from all patients and controls. All participants signed written informed consent before joining the study, agreeing that the results of the study might be presented or published for scientific reasons.

### Physical examination and laboratory tests

Before the physical examination, all participants were required to fast for 8–10 h. After recording medical histories and anthropometric characteristics, blood pressure was measured. After 10 min of rest, blood pressure was measured three times at 2-min intervals on the subject’s upper arm using a mercury sphygmomanometer (YuYue Medical Equipment & Supply CO., LTD., Jiangsu Province, China). The mean of the three readings was used for data analysis. The International Collaborative Study on Hypertension in Blacks (ICSHIB) protocol was used to determine anthropometric parameters including height, weight, and hip and waist circumference (WC) (12). Body mass index (BMI) was calculated by dividing body weight in kilograms by height in meters squared. The waist-to-hip circumference was used to compute the waist-to-hip ratio (WHR). We used bioelectrical impedance (BIA-101; RJL Systems, Shenzhen, China) to measure the percentage of body fat (FAT%).

Venous blood was collected in tubes after 10–12 h of overnight fasting. The tubes were centrifuged, and plasma aliquots were collected and stored at –80°C until analysis. TG, TC, LDL-C, HDL-C, and hsCRP were determined by enzymatic colorimetric method with a HITACHI 7600 series autoanalyzer (HITACHI, Tokyo, Japan). The white blood cell count was measured using a Mindray BC-3000 Hematology Analyzer (Mindray, Shenzhen, China). FBG was assayed using the glucose oxidase method. HbA1c was determined by an ion exchange HPLC method. Plasma NO concentrations were determined by the Griess method (13). Circulating sortilin, adiponectin (ADI), ET-1, and interleukin-6 concentrations were determined by commercial ELISA kits following the manufacturer’s protocol (Jingmei Engineering, Jiangsu Province, China). For all kits, the inter-assay and intra-assay coefficients of variation were < 12 and < 8%, respectively. The minimum detectable concentration for sortilin was 0.1 ng/ml.

### Vascular ultrasound measurement

High-resolution B-mode ultrasonography (Philips Epiq7 system; Philips, Amsterdam, Netherlands) was used to measure the carotid intima-media thickness (cIMT). A single radiologist performed all of the cIMT measurements. The distance between the lumen-intima interface and media-adventitia interface of the far wall was defined as the cIMT. The intimal-medial thickness was estimated as the mean of measurements made from 0.5 to 2 cm below the carotid bifurcation of the common carotid artery on each side. IMT measurements were taken at the thickest point. Plaque was defined as having an IMT ≥ 1.5 mm or a focal protrusion into the lumen that was at least 50 percent thicker than the neighboring intima-media complex (14).

### Statistical analysis

All statistical analyses were conducted using the Windows version of SPSS 26.0 (SPSS Inc., Chicago, Illinois, USA) and R software (version 4.1.3; R Foundation for Statistical Computing, Vienna, Austria). The Kolmogorov–Smirnov test was used to determine the normality distribution of continuous variables. The results are expressed as the mean ± standard deviation (SD) or median (25th–75th percentiles). Categorical variables were expressed as frequencies and percentages. Non-normally distributed data were converted by a logarithmic transformation before analysis. A paired *t*-test or an unpaired *t*-test was used for comparisons among groups. Differences among the groups were tested using analysis of variance (ANOVA). Correlational analyses were performed using Pearson’s or Spearman’s correlation tests. Multiple regression analysis was performed to correct the effects of the covariates and evaluate the independent factors. Logistic regression analysis was performed to determine independent predictors of EH and subAS in EH. Restricted cubic splines were used to examine the correlation between plasma sortilin levels and the risk of EH. For all analyses, *P* < 0.05 was considered statistically significant.

## Results

### Clinical characteristics of study participants

The clinical features of the 336 individuals are detailed in Table 1. The EH and NT groups were matched for age and sex, and all of the subjects did not take antihypertensive drugs at the initial visit. There were no statistically significant differences between EH and NT subjects with respect to age, sex, FAT%, TG, HDL-C, FBG, HbA1c, WBC, and hsCRP. Compared to the NT group, the EH group had significantly higher BMI, WHR, SBP, DBP, TC, LDL-C, IL-6, and ET-1 levels, and lower NO levels (*P* < 0.001 or *P* < 0.05 in all comparisons).

**TABLE 1:** Clinical characteristics of study subjects.

Variable	EH (*n* = 186)	NT (*n* = 150)	P^a^	EH + subAS (*n* = 93)	EH-subAS (*n* = 93)	P^b^
**Demographic characteristics**						
Male/female	79/107	70/80	NS	49/44	30/63	0.005
Age (yr)	59.96 ± 12.50	58.95 ± 10.50	NS	64.98 ± 12.02	54.94 ± 10.90	<0.001
BMI (kg/m^2^)	24.76 ± 3.61	24.03 ± 2.74	0.039	24.88 ± 3.63	24.64 ± 3.60	NS
WHR	0.91 ± 0.05	0.87 ± 0.08	<0.001	0.92 ± 0.05	0.91 ± 0.05	NS
FAT%	30.16 ± 5.14	29.22 ± 5.46	NS	30.63 ± 5.45	29.68 ± 4.79	NS
SBP (mmHg)	170.30 ± 19.07	117.55 ± 10.83	<0.001	171.17 ± 18.16	169.43 ± 20.00	NS
DBP (mmHg)	90.45 ± 11.97	70.58 ± 8.01	<0.001	89.48 ± 11.02	91.41 ± 12.84	NS
**Laboratory examination**						
TG (mmol/L)^a^	1.66 (1.13,2.29)	1.50 (1.23,2.28)	NS	1.78 (1.19,2.34)	1.53 (1.02,2.14)	NS
TC (mmol/L)	5.12 ± 1.14	4.78 ± 1.13	0.008	5.23 ± 1.25	5.00 ± 1.04	NS
HDL-C (mmol/L)	1.19 ± 0.25	1.25 ± 0.31	NS	1.16 ± 0.24	1.22 ± 0.26	NS
LDL-C (mmol/L)	2.79 ± 0.88	2.49 ± 0.78	0.001	2.83 ± 0.92	2.73 ± 0.84	NS
FBG (mmol/L)	5.59 ± 0.49	5.47 ± 0.60	NS	5.66 ± 0.45	5.52 ± 0.50	0.053
HbA1c (%)	5.59 ± 0.51	5.54 ± 0.57	NS	5.64 ± 0.53	5.55 ± 0.50	NS
hsCRP (mg/L)^a^	2.4 (1.0,3.9)	2.3 (1.2,3.4)	NS	2.4 (1.1,3.8)	2.7 (1.0,4.2)	NS
WBCx 10^9^/L	6.26 ± 1.58	5.93 ± 1.54	0.051	6.25 ± 1.40	6.27 ± 1.74	NS
IL-6 (pg/ml)	37.11 ± 6.20	26.51 ± 6.46	<0.001	36.89 ± 6.67	37.34 ± 5.71	NS
NO (μmol/L)^a^	3.36 (1.86,5.29)	6.18 (4.40,9.36)	<0.001	3.32 (1.73,5.00)	3.37 (2.01,5.73)	NS
ET-1 (pg/ml)	119.68 ± 21.21	98.09 ± 22.10	<0.001	115.89 ± 22.55	123.47 ± 19.17	0.015
ADI (μg/ml)	19.55 ± 4.06	26.40 ± 4.37	<0.001	19.49 ± 4.20	19.61 ± 3.95	NS
Sortilin (ng/ml)	8.10 ± 1.82	6.37 ± 1.52	<0.001	8.42 ± 1.75	7.79 ± 1.84	0.019
**Vascular ultrasound measurement**						
cIMT (mm)	0.84 ± 0.16	−	−	0.92 ± 0.15	0.76 ± 0.12	<0.001
With carotid plaque	86 (46.2%)	−	−	86 (92.5%)	−	−

Data are expressed as the mean with SD and median (interquartile range).

P-value for difference between the groups was calculated from Student’s t-test.

^a^Natural logarithmic transformation was used in analysis.

P^a^ between the EH and NT groups.

P^b^ between the subAS and non-subAS subgroups.

BMI, body mass index; WHR, waist/hip ratio; SBP, systolic blood pressure; DBP, diastolic blood pressure; hsCRP, high-sensitivity C-reactive protein; WBC, white blood cell; cIMT, carotid intima–media thickness; HDL-C: High-density lipoprotein-cholesterol; LDL-C, low-density lipoprotein cholesterol; TG, triglyceride; TC, total cholesterol; HbA1c, hemoglobin A1c; FBG, fasting blood glucose; ET-1, endothelin-1; ADI, adiponectin; IL, interleukin.

Compared with patients without subclinical carotid atherosclerosis, patients with subclinical carotid atherosclerosis were likely to be male and older (64.98 ± 12.02 years vs. 54.94 ± 10.90 years, *p* < 0.001), and showed lower ET-1 levels (*P* < 0.05).

### Plasma sortilin and adiponectin levels in the study participants

**FIGURE 1:**
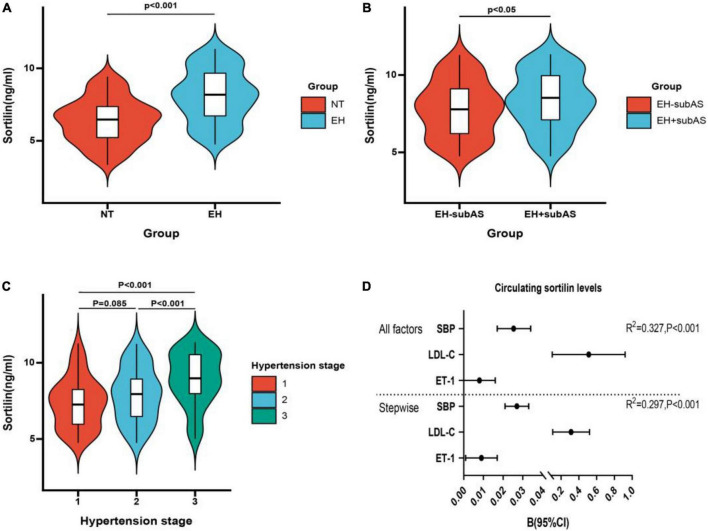
Concentrations of circulating sortilin in study population. **(A)** Circulating sortilin levels in the EH and NT groups. **(B)** Circulating sortilin levels in hypertensive subjects with subclinical carotid atherosclerosis (EH + subAS) or without subclinical carotid atherosclerosis (EH-subAS). **(C)** Comparison of the concentration of sortilin in different hypertension stages in the EH group. **(D)** All factors and stepwise multiple regression analyses of circulating sortilin in all study populations. The circles correspond to the regression coefficients β, and the error bars indicate the 95% confidence interval (CI) of β.

We observed no difference in plasma sortilin levels between men and women. Plasma sortilin levels were significantly increased in the EH group compared with the NT group (8.10 ± 1.82 ng/ml vs. 6.37 ± 1.52 ng/ml, *p* < 0.001) (Figure 1A). Patients with subclinical carotid atherosclerosis exhibited higher sortilin concentrations than the EH-subAS groups (8.42 ± 1.75 ng/ml vs. 7.79 ± 1.84 ng/ml, *P* < 0.05) (Figure 1B). Subgroup analysis was performed in the EH group according to hypertension stage. Plasma sortilin levels were significantly higher in stage 3 hypertension than in stage 1 hypertension and stage 2 hypertension (8.96 ± 1.73 ng/ml vs. 7.37 ± 1.63 ng/ml, *P* < 0.001; 8.96 ± 1.73 ng/ml vs. 7.89 ± 1.75 ng/ml, *P* < 0.001) (Figure 1C).

Plasma ADI concentrations were markedly lower in the EH group than in the NT group (19.55 ± 4.06 μg/ml vs. 26.40 ± 4.37 μg/ml, *P* < 0.001) (Table 1).

### The association of circulating sortilin with anthropometric and biochemical parameters in the study population

**FIGURE 2:**
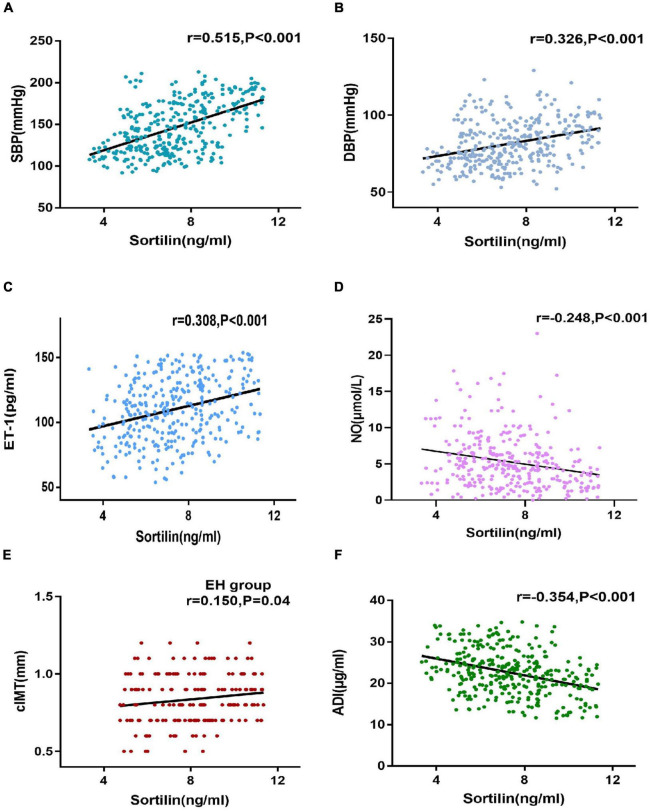
Correlations of sortilin levels and parameters. **(A–F)** Correlations of sortilin levels and SBP **(A)**, DBP **(B)**, ET-1 **(C)**, NO **(D)**, and ADI **(F)** in the general population. Correlations of sortilin levels and cIMT **(E)** in the EH group.

We next investigated the relationship between plasma sortilin levels and various parameters, and the results are shown in Table 2. Plasma sortilin concentrations were positively correlated with SBP (*r* = 0.515, *p* < 0.001) (Figure 2A), DBP (*r* = 0.326, *p* < 0.001) (Figure 2B), TG (*r* = 0.150, *p* = 0.006), TC (*r* = 0.192, *p* < 0.001), LDL-C (*r* = 0.252, *p* < 0.001), WBC (*r* = 0.146, *p* = 0.007), hsCRP (*r* = 0.129, *p* = 0.018), IL-6 (*r* = 0.257, *p* < 0.001), and ET-1 (*r* = 0.308, *p* < 0.001) (Figure 2C) but negatively correlated with NO (*r* = −0.248, *p* < 0.001) (Figure 2D) and ADI (*r* = −0.354, *p* < 0.001) (Figure 2F). In the EH group, plasma sortilin concentrations were positively correlated with cIMT (*r* = 0.150, *p* = 0.04) (Figure 2E). We then performed multiple stepwise regressions to identify variables independently associated with plasma sortilin, and the results revealed that SBP, LDL-C, and ET-1 were independent predictors of plasma sortilin levels (Figure 1D and Table 2). The multiple regression equation was Y_*Sortilin*_ = 1.519 + 0.027X_*SBP*_ + 0.009X_*ET*–1_ + 0.312X _*LDL*–*C*_ (*R*^2^ = 0.297, *P* < 0.001).

**TABLE 2:** Linear regression analysis of variables associated with circulating sortilin levels in the study population.

Variable	Simple		Multiple	
		
	*r*	*P*-value	β	*P*-value
Age (yr)	–0.059	0.284		
BMI (kg/m^2^)	0.105	0.055		
WHR^a^	0.105	0.054		
FAT%	0.106	0.052		
SBP (mmHg)	0.515	<0.001	0.027	<0.001
DBP (mmHg)	0.326	<0.001		
TG (mmol/L)^a^	0.150	0.006		
TC (mmol/L)	0.192	<0.001		
HDL-C (mmol/L)	–0.049	0.368		
LDL-C (mmol/L)	0.252	<0.001	0.312	0.003
FBG (mmol/L)	–0.037	0.501		
HbA1c%	–0.082	0.134		
WBCx 10^9^/L	0.146	0.007		
hsCRP (mg/L)^a^	0.129	0.018		
IL-6 (pg/ml)	0.257	<0.001		
NO (μmol/L)^a^	–0.248	<0.001		
ET-1 (pg/ml)	0.308	<0.001	0.009	0.023
ADI (μg/ml)	–0.354	<0.001		

In multiple linear regression analysis, values included for analysis were age, BMI, WHR, FAT%, SBP, FBG, HbA1c, TC, HDL-C, LDL-C, TG, NO, ET-1, IL-6, hsCRP, and ADI.

^a^Spearman correlation tests were used in analysis.

BMI, body mass index; WHR, waist/hip ratio; SBP, systolic blood pressure; DBP, diastolic blood pressure; hsCRP, high-sensitivity C-reactive protein; WBC, white blood cell; cIMT, carotid intima–media thickness; HDL-C: High-density lipoprotein-cholesterol; LDL-C, low-density lipoprotein cholesterol; TG, triglyceride; TC, total cholesterol; HbA1c, hemoglobin A1c; FBG, fasting blood glucose; ET-1, endothelin-1; ADI, adiponectin; IL, interleukin.

### Association of plasma sortilin levels with the risk of essential hypertension and carotid atherosclerosis in essential hypertension

We performed multivariable logistic regression analysis to further investigate the association between sortilin and the occurrence of EH and carotid atherosclerosis in EH. Plasma sortilin was found to be an independent impact factor for hypertension (OR: 1.86, 95% CI: 1.56–2.20, *P* < 0.001), even after controlling for age, sex, BMI, WHR, FAT%, glucose homeostasis, and lipid profile (Table 3). Subclinical atherosclerosis was present in 93 (50%) hypertensive patients. After controlling for anthropometric variables, age, sex, BMI, BP, glucose homeostasis, and lipid profile, multivariable logistic regression analysis showed significant independent associations of sortilin with subclinical atherosclerosis (OR: 1.33, 95% CI: 1.07–1.66, *P* < 0.05) in the EH group (Table 3).

**TABLE 3:** Odds ratio of circulating sortilin levels with EH and subAS in EH by multivariate logistic regression analysis.

Model adjustment	subAS in EH			EH		
		
	OR	95%CI	*P*	OR	95%CI	*P*
Model 1	1.34	1.11–1.62	0.002	1.82	1.56–2.12	<0.001
Model 2	1.36	1.13–1.65	0.002	1.81	1.56–2.11	<0.001
Model 3	1.37	1.13–1.66	0.001	1.81	1.55–2.11	<0.001
Model 4	1.37	1.13–1.66	0.001	1.80	1.53–2.11	<0.001
Model 5	1.34	1.11–1.63	0.003	1.81	1.54–2.13	<0.001
Model 6	1.35	1.11–1.64	0.003	1.83	1.55–2.15	<0.001
Model 7	1.32	1.08–1.62	0.007	1.86	1.56–2.20	<0.001
Model 8	1.33	1.07–1.66	0.011	−	−	−

Results of multivariate logistic regression analysis are presented as the odds ratio (OR); Model 1, adjusted for age; model 2, adjusted for age and sex; model 3, adjusted for age, sex, and BMI; model 4, adjusted for age, sex, BMI, and WHR; model 5, adjusted for age, sex, BMI, WHR, and FAT%; model 6, adjusted for age, sex, BMI, WHR, FAT%, HbA1C%, and FBG; model 7, adjusted for age, sex, BMI, WHR, FAT%, HbA1C%, FBG, and lipid profile; model 8, adjusted for age, sex, BMI, WHR, FAT%, HbA1C%, FBG, lipid profile, and blood pressure. Lipid profile includes TC, TG, LDL-C, and LDL-C.

**FIGURE 3:**
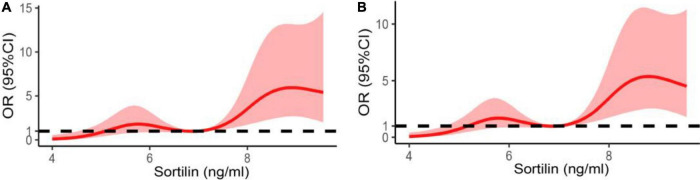
Restricted spline curve of the plasma sortilin levels and the odds ratio of hypertension. **(A)** The restricted spline curve of univariable logistic regression model. **(B)** The restricted spline curve of multivariable logistic regression model. Adjusted for age, sex, BMI, WHR, FAT%, FBG, HbA1c, TG, TC, LDL-C, and HDL-C.

Restricted cubic splines were also used to detect the association between the plasma sortilin level and the risk of EH. The results demonstrated that the higher the plasma sortilin level, the higher the incidence of EH (Figure 3).

## Discussion

In this study, we demonstrated that newly diagnosed hypertensive patients had higher circulating sortilin concentrations than age and sex-matched healthy controls, and the concentrations of sortilin were further elevated in hypertensive patients with subclinical carotid atherosclerosis compared to those without. Moreover, we detected an independent association of sortilin with hypertension as well as subclinical atherosclerosis in hypertension.

Recently, more studies have focused on the relationship between sortilin and cardiovascular disease. Serum sortilin was elevated in patients with coronary artery disease and carotid artery stenosis, and correlated with the severity of coronary artery disease and carotid plaque burden (8, 15). It has been recently reported that sortilin changes sphingolipid/ceramide homeostasis and initiates the endothelial cell signaling pathway, resulting in increased production of reactive oxygen species (ROS), leading to increased arterial blood pressure (9, 16). However, in a cohort study of 1,173 low- to intermediate-risk chest-pain patients, plasma sortilin was not associated with CAD severity and coronary calcium score, and sortilin did not improve existing risk stratification in the clinical environment (10). It is still controversial whether sortilin can be used as a biomarker of cardiovascular disease. Furthermore, previous studies did not exclude the effects of comorbidities and drugs on the experimental results. Therefore, to avoid these shortcomings, we investigated the association of sortilin with hypertension and subclinical atherosclerosis in newly diagnosed hypertensive patients.

Hypertension is a complex disease with a multifaceted pathogenesis. It is considered to be the result of disturbances in a number of neural, renal, hormonal, and vascular mechanisms, in which impairment of constriction and relaxation of vessels is the main pathophysiologic characteristic (17). Research has found that macrophages lacking sortilin secreted decreased levels of IL-6 and TNF-α, reducing vascular inflammation and vascular remodeling (18). In our study, circulating sortilin, IL-6, and hsCRP levels increased in the EH group compared with the NT group. Moreover, correlation analysis suggested that sortilin was closely related to IL-6, and hsCRP, suggesting that elevated plasma sortilin levels may be related to endothelial cell damage and inflammatory cytokine release in hypertensive patients.

Hypertension is accompanied by chronic vascular inflammation and endothelial dysfunction, which interact with each other (19). In the present study, circulating ET-1 levels increased and NO levels decreased in the EH group compared with the NT group. Correlation analysis demonstrated that sortilin levels were significantly positively correlated with ET-1 levels and negatively correlated with NO levels in the general population. The imbalance between ET-1 and NO produced by vascular endothelial cells can cause endothelial dysfunction, elevated proinflammatory events, as well as increased oxidative stress, thus leading to the development of hypertension (16, 20, 21). It has been reported that sortilin induces ROS overproduction and endothelial dysfunction by activating the acid sphingomyelinase (ASMase)/sphingosine-1-phosphate (S1P) pathway (9). The signaling pathways linking S1P and ROS generation could be a bridge between cardiovascular and immune systems (16), which might be involved in the pathogenesis of hypertension, especially given that sortilin regulates the secretion of proinflammatory cytokines (IL-6 and interferon-γ) by immune cells (18). Furthermore, we found that the incidence of EH increases with higher plasma sortilin levels by using multivariate logistic regression. The mechanism of the elevation of circulating sortilin in patients with cardiovascular disease is still unclear. Previous research speculated that sortilin may be released into the blood by activated platelets (22). While this study did not allow us to deduce the causal relationship between the elevation of sortilin and hypertension, combined with the previous role of sortilin in CVDs, we consider that sortilin may contribute to high blood pressure by affecting the function and structure of blood vessels.

Long-term hypertension causes arterial damage and progressively leads to atherosclerosis, all of which are associated with an increased risk of cardiovascular complications. Therefore, exploring a biomarker associated with subAS in EH is crucial in preventing cardiovascular disease and target organ damage. In the current study, hypertensive patients with subAS were older, had a higher proportion of men, and had considerably higher plasma sortilin levels. Despite adjustment for cardiovascular risk factors such as blood pressure and lipid profile, sortilin was still an independent risk factor for subclinical carotid atherosclerosis in EH patients. The potential mechanism to explain this association is that sortilin acts as an energetic regulator of lipid metabolism, plays a pathogenic role in cardio-metabolic diseases (23). An original study discovered that sortilin-mediated VLDL secretion led to a high concentration of plasma LDL-C, which invades subendothelial space (24). Sortilin in macrophages continuously phagocytoses invading lipid components to increase the creation of foam cells and the development of atherosclerotic plaques (4). In line with previous findings (25, 26), we found a significant positive correlation between plasma sortilin and LDL-C, TG, and TC levels. Notably, the relationship between sortilin and plasma lipids is still controversial. Sortilin levels have been reported to be negatively correlated with TG, TC, and LDL-C levels in patients with type 2 diabetes (27). Another study found that statin therapy decreased plasma sortilin levels, but they did not observe a correlation between serum sortilin levels and lipid parameters (28). More research is needed in the future to explore the interaction between sortilin and blood lipids in different pathological states.

We also observed a correlation between plasma concentrations of sortilin and cIMT. In contrast, it has been reported that elevated serum sortilin levels had no relation with cIMT in patients with cardiovascular risk factors, which was inconsistent with our results (25). The inconsistent conclusions on the association of sortilin and atherosclerosis in different patients stimulate further studies to examine the impact of sortilin on atherosclerosis in humans and animals.

In addition, our data revealed that sortilin was associated with adipokines. In the present study, plasma sortilin levels were significantly negatively correlated with ADI levels in hypertensive patients. ADI regulates blood pressure by promoting the production of NO and inhibiting vasoconstriction, and prevents atherosclerosis through anti-inflammatory effects and reducing ROS production (29). It also promotes fatty acid oxidation, inhibits lipid synthesis, and reduces blood lipids (30). Hagita et al. found that knockout of SORT1 increased plasma ADI levels in high-fat-fed female LDLR−/− mice, while reducing mouse body weight and plasma total cholesterol levels (31). Therefore, we speculate that the effects of sortilin on vascular endothelium, metabolism, and inflammation may be partially achieved through its association with ADI. Further research is needed to investigate whether there is an interaction between sortilin and ADI.

The concentration of circulating sortilin was different in various studies. Previous studies found that sortilin concentrations ranged from 0.3 to 5 ng/ml (6, 7, 25); Goettsch et al. found that sortilin concentrations were higher in older men, with a median of 77.5 ng/ml (26); The mean plasma sortilin concentration in our study was 7.24 ng/ml (range 3.34–11.34 ng/ml). These differences may be caused by different races, study populations, or different ELISA kits and protocols recommended by the manufacturer.

Our study has several limitations: (1) cross-sectional research cannot show a causal relationship between variables, (2) our study was a single-center observational study, and the sample size was relatively small, which has the potential for selection bias that might affect outcomes, (3) although our study included newly diagnosed hypertensive patients, we did not evaluate other potential factors that may affect the results, such as non-alcoholic fatty liver and neurological diseases, and (4) due to the limitations of our experimental conditions, cIMT was not collected in our healthy control group. More studies are needed regarding the possible role of plasma sortilin levels in essential hypertension and atherosclerosis.

## Conclusion

Our study demonstrates that circulating sortilin levels are elevated in hypertensive patients, and positively correlate with EH and subAS in EH. The role of sortilin in hypertension and atherosclerosis and its potential clinical application require further investigation.

## Data availability statement

The raw data supporting the conclusions of this article will be made available by the authors, without undue reservation.

## Ethics statement

The studies involving human participants were reviewed and approved by the Human Research Ethics Committee at the Second Affiliated Hospital of Chongqing Medical University. The patients/participants provided their written informed consent to participate in this study.

## Author contributions

RL, DK, and XC: research idea, study design, and writing manuscript. XC, YJ, and TG: sample and data acquisition. RL, CL, and XC: sample analysis. XC and YD: statistical analysis. All authors contributed to the article and approved the submitted version.

## Abbreviations

ED: endothelial dysfunction
subAS: subclinical carotid atherosclerosis
ET-1: endothelin-1
NO: nitric oxide
ADI: adiponectin
IL-6: interleukin-6
BMI: body mass index
WHR: waist/hip ratio
hsCRP: high-sensitivity C-reactive protein
HDL-C: High-density lipoprotein-cholesterol
LDL-C: low-density lipoprotein cholesterol
TG: triglyceride
HbA1c: Hemoglobin A1c
FBG: fasting blood glucose
cIMT: carotid intima–media thickness.
